# Genotypic and Phylogenetic Insights on Prevention of the Spread of HIV-1 and Drug Resistance in “Real-World” Settings

**DOI:** 10.3390/v10010010

**Published:** 2017-12-28

**Authors:** Bluma G. Brenner, Ruxandra-Ilinca Ibanescu, Isabelle Hardy, Michel Roger

**Affiliations:** 1 McGill University AIDS Centre, Lady Davis Institute for Medical Research, Montreal, QC H3T 1E2, Canada; ribanescu@jgh.mcgill.ca; 2 Département de Microbiologie et d’Immunologie et Centre de Recherche du Centre Hospitalier de l’Université de Montréal (CHUM), Montreal, QC H2X 0A9, Canada; isabelle.hardy.chum@ssss.gouv.qc.ca (I.H.); michel.roger.chum@ssss.gouv.qc.ca (M.R.)

**Keywords:** HIV-1/AIDS, phylogenetics, HIV drug resistance, genotyping, antiretroviral therapy, treatment-as-prevention

## Abstract

HIV continues to spread among vulnerable heterosexual (HET), Men-having-Sex with Men (MSM) and intravenous drug user (IDU) populations, influenced by a complex array of biological, behavioral and societal factors. Phylogenetics analyses of large sequence datasets from national drug resistance testing programs reveal the evolutionary interrelationships of viral strains implicated in the dynamic spread of HIV in different regional settings. Viral phylogenetics can be combined with demographic and behavioral information to gain insights on epidemiological processes shaping transmission networks at the population-level. Drug resistance testing programs also reveal emergent mutational pathways leading to resistance to the 23 antiretroviral drugs used in HIV-1 management in low-, middle- and high-income settings. This article describes how genotypic and phylogenetic information from Quebec and elsewhere provide critical information on HIV transmission and resistance, Cumulative findings can be used to optimize public health strategies to tackle the challenges of HIV in “real-world” settings.

## 1. Introduction

The HIV/AIDS pandemic has had a devastating global impact, eroding public health, social and economic infrastructures worldwide [1,2]. Whereas concentrated subtype B epidemics prevail among men having sex with men (MSM) and intravenous drug users (IDU) in the Western world [3,4], generalized heterosexual (HET) epidemics in Africa and Asia have diversified into an increasingly complex disease, with nine major viral subtypes and more than 70 circulating recombinant forms [1,2]. Migration and globalization patterns have contributed to the introduction and crossover of epidemics in America and Europe, wherein non-B viral subtypes now account for 20% and 60% of new infections in North America and Europe, respectively [5,6].

Outstanding advances in antiretroviral therapy (ART) have transformed HIV/AIDS from a deadly disease to a treatable and potentially preventable disease [7,8]. Since 2010, expanded ART coverage to 20.9 million of the 36.7 million persons living with HIV has resulted in 11% and 47%, declines in new infections among adults and children by respectively (www.unaids.org) [9,10,11]. The goal of ART has shifted from addressing the health benefits of the individual to control of the global HIV-1 pandemic [9,10,11]. The World Health Organization has advanced the “Getting to Zero” policy by 2030 with the “90-90-90” target for 2020, wherein 90% of HIV-infected persons are aware of their status, 90% of infected persons receive immediate ART and 90% of treated individuals achieve long-term viral suppression.

First-line ART in low- and middle-resource settings are largely limited to combination regimens containing two-nucleoside reverse transcriptase (RT) inhibitors (NRTIs) and a non-nucleoside reverse transcriptase inhibitor (NNRTI) [12,13,14]. In resource-rich settings, recent guidelines recommend the combination of two NRTIs with an integrase strand transfer inhibitor (INSTI). The superiority of triple combinations that include an INSTI over an NNRTI or a boosted protease inhibitor (PI) is based on improved tolerability, better drug-drug interactions dosing and higher genetic barrier to resistance [14,15].

HIV drug resistance remains an emerging threat to the long-term success of HIV treatment programs and epidemic control [16]. As more individuals are started on ART, treatment goals will require more robust and durable first-line regimens. Drug resistance testing programs can identify pre-existing and acquired resistance mutations, customizing and optimizing treatment options and clinical management over time [17,18,19,20,21]. Drug resistance testing is recommended at diagnosis, before ART initiation and at the time of virological failure, where possible.

While the role of ART in reducing HIV transmission is undisputed [22], there has been a resurgence of HIV epidemics among MSM and IDU in the Western world and growing rates of transmitted resistance in the Third world. This underscores the need for strong surveillance systems to understand drivers of regional epidemics and improve antiviral treatment and prevention strategies in “real-world” settings [3,23,24,25,26,27,28]. Genotypic drug resistance programs can also be used to provide salient information on the introduction and spread of viral subtypes in different at-risk groups at a population-level networks [27,29,30,31,32,33,34,35,36,37,38].

Phylogenetic analyses of sequence data from national genotyping programs provide an evidence-based framework to deduce the evolutionary interrelationship of viral strains and identify transmission clusters. Phylogenetic surveillance can clarify factors underlying HIV transmission and better inform public health policy [29,30,39,40,41,42,43]. A better understanding of the mechanisms underlying transmission networks may help balance and maximize the efficiency of Treatment-as Prevention strategies.

Mark A. Wainberg was a pioneer in the field of HIV drug resistance, defining signature resistance mutational patterns and related viral fitness costs implicated in the evolution of drug resistance in different HIV-1 viral subtypes. He was also instrumental in advocating for expanded access of the best antiretroviral drugs in Africa [14,15,44]. In this tribute, we provide an update on current knowledge on genotypic and phylogenetic inferences on HIV transmission and drug resistance in Quebec and elsewhere. Cumulative phylogenetic and genotypic findings provide important insights to inform public health interventions in “real-world” settings.

## 2. Results and Discussion

### 2.1. Phylogenetic Inferences on the Geographic Introduction and Spread of HIV

The high genetic variability of HIV-1 is a direct consequence of the high replicative rate of the virus, the high error rates of the viral reverse transcriptase (RT) enzyme and viral plasticity under selective host and drug pressure [17,45]. Although HIV continually evolves and adapts in infected hosts, there are stringent bottlenecks leading to transmission of a single monophyletic variant to most (>95%) newly-infected persons. Phylogenetic analysis can track the linkage of viral variants at a population-level, providing a molecular epidemiological framework for surveillance of HIV transmission dynamics. Moreover, phylogenetics can map the geographic introduction and domestic expansion of viral subtypes in different regional settings [29,31,34,46].

**Figure 1:**
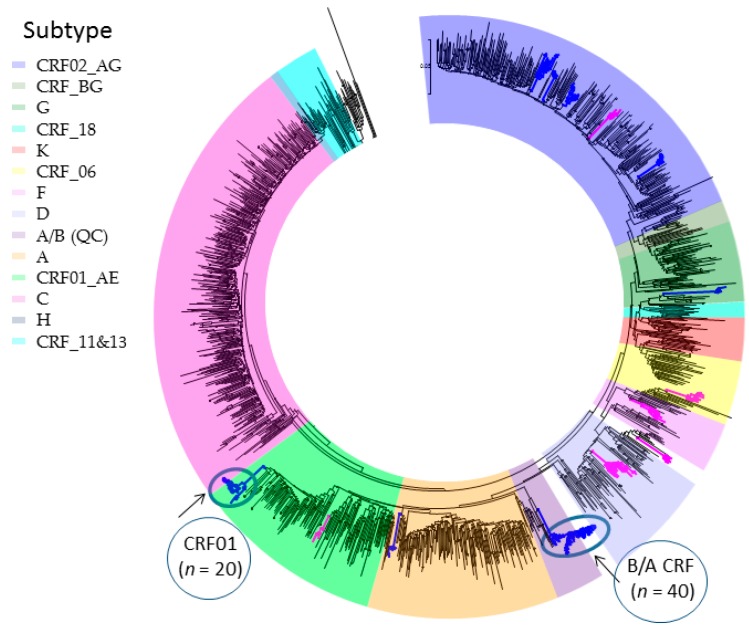
Phylogenetic surveillance of the introduction and spread of non-B subtypes in Quebec [47]. First genotypes of new non-B subtypes (*n* = 1395) reflects the diversity of the African pandemic. Transmission clusters of 5+ members reveal onward spread among Men having Sex with Men (MSM, blue) and Heterosexual (HET, pink) groups. Large clusters (20+ members) are circled.

The global diversity of major non-B viral subtypes (A, C, D, F, G, H, J, K) and circulating recombinant forms (CRFs) is reflected in non-B subtype epidemic in Quebec (Figure 1). The non-B viral subtypes into Quebec (18% of the provincial epidemic) has arisen through immigration of individuals from francophone countries in Central and West Africa, at the epicenter of the global pandemic. Overall, subtype C, subtype CRF02_AG/G and subtype A/CRF01_AE represent 29%, 28% and 24% of new infections. Subtypes D, F and other CRFs contribute to 6.4%, 2.5% and 10% of the remaining non-B subtype epidemic. Phylogenetics reveal an infrequent onward domestic spread of non-B subtype strains within the province. Two major clusters, circled in Figure 1, depict a Quebec founder subtype A/B CRF (*n* = 40 members) strain and a subtype CRF01_AE (*n* = 21 members) cluster spreading among MSM.

As illustrated, phylogenetic surveillance can provide important insights on the geographic entry, expansion and crossover of epidemics among HET, MSM and IDU populations [48,49,50,51,52,53]. This can assist in the design of public health interventions, tailored to at-risk vulnerable groups.

### 2.2. Phylogenetic Analysis of Transmission Clustering

Despite the remarkable global spread of HIV, the relative risk of acquiring infection per exposure is low; with risk estimates of 0.1%, 0.6% and 1.4% per contact for HET, IDU and MSM route of transmission, respectively [54,55]. Cumulative findings in multiple cohorts, across subtypes, show a bottleneck in HIV-1 transmission wherein a single founder establishes HIV infection in any given newly infected partner despite high diversity of viral quasispecies in infected source partners [56,57,58,59].

Drug resistance testing programs provide viral polymerase *pol* sequence datasets, covering a relatively large proportion of the infected populations at a state/provincial, national, or regional scale [27,28,29,31,35,43,49]. HIV-1 transmission chains are determined based on sequence similarity. Typically, a single (*pol*) gene sequence from national drug resistance testing program are obtained from each newly HIV-infected person in the population. Phylogenetic trees of viral sequences can be reconstructed using Neighbor-joining, Maximum Likelihood, Bayesian, or Gap approaches [33,49,60]. Transmission clustering of linked viral sequences is generally based on strong bootstrap support (with cutoffs generally exceeding 0.90), short within-cluster genetic distance (0.01–0.05 substitutions/site) or posterior probabilities [33,49].

Cumulative analyses of datasets with high genotypic coverage (>30% of the infected population) have implicated transmission clustering as a major driver in the spread of concentrated HIV-1 epidemics among MSM and IDU populations [29,31,34]. Unfortunately, drug resistance programs are not available or affordable in the majority of low- and middle-income countries ART [61,62]. As such, analysis of epidemics principally driven by viral spread in HET populations such as Africa, is far less understood.

### 2.3. Phylogenetic Surveillance of Transmission Dynamics of the Quebec MSM Epidemic

In Quebec, the provincial genotyping program has 27,487 *pol* sequences from 9785 persons, capturing driving forces implicated in the spread of subtype B epidemics in MSM (65%) and IDU (15%) and non-B subtype HET populations (20%) (2002–2016) [27]. Overall, IDU epidemics have declined in the post-ART era. In contrast, the high frequency of viral co-clustering (66%) among newly-diagnosed and ART-naïve MSM infer frequent re-transmissions among newly-infected persons, often unaware of their HIV status.

**Figure 2:**
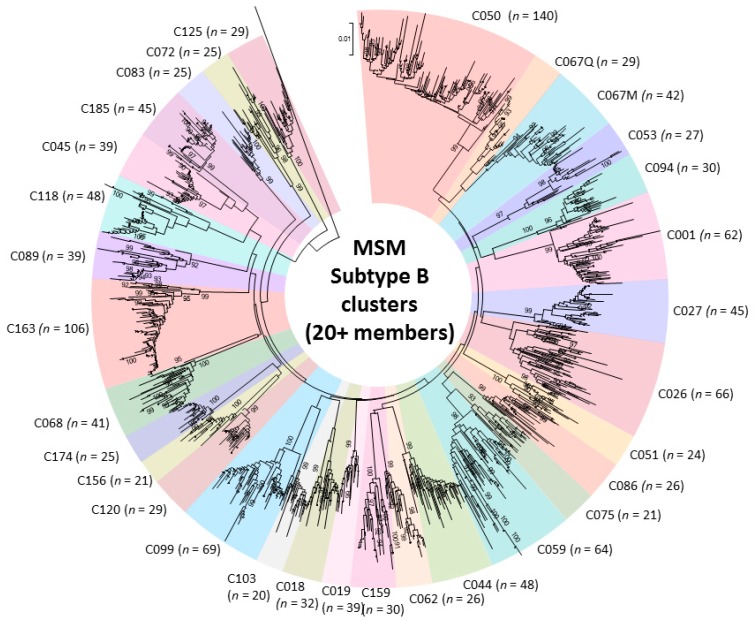
Phylogenetic tree of 31 subtype B large cluster (20+ members) transmission networks fueling onward spread of the epidemic among Men having Sex with Men (MSM) (2004–2015).

Genotypic analysis across multiple cohorts has shown the role of recent infection as a driver of onward spread of the provincial epidemic [27,29,30,39,41]. Phylogenetic linkage studies show an increasingly large role of large cluster networks implicated in viral spread, as well as drug-resistant sub-epidemics [27,29,30,39,41]. Half of the epidemic in MSM can be ascribed to “dead-end” transmissions or small clusters of 2–4 individuals expanding over median 4.75 month intervals. The remaining half of the epidemic can be ascribed to increasingly large cluster networks. Phylodynamics reveal that thirty viral strains have sustained the epidemic in MSM. These large cluster networks, involving 20 to 140 persons have risen from 13%, 25%, to 42% of new infections over the 2004–2007, 2008–2011 and 2012–2015 periods, respectively (Figure 2) [27].

Integration of phylogenetic, virological and behavioral information show large 20+ clusters outbreaks as compared to singleton transmissions are associated with primary/recent stage infection, younger populations (under 30 years of age) and having more than 5 partnerships prior to PHI (Odds-ratios of 3.7, 3.3 and 1.4, respectively (App. 2) [34].

Overall, 1.7% of viral species (*n* = 60) have contributed to large cluster networks (10+ members), sustaining the growth of the epidemic among MSM over the last decade. Although it is possible that this can arise partially because of random, stochastic events reflective of the low frequency at which the HIV-1 is transmitted, it may also indicate that there is a selective advantage for variants with certain genotypic and phenotypic features. Our recent findings used in vitro drug selections show a selection bias for large cluster viral variants showing higher replicative fitness under selective drug pressure [63]. Other lines of evidence to support selective transmission of viral species include the observations that (1) early virus populations are less genetically diverse than the source-virus quasispecies populations; (2) viruses present early in infection generally use the chemokine receptor type 5 (CCR5) co-receptor rather than the C-X-C chemokine receptor type 4 (CXCR4) variant for entry; and (3) early viruses are selectively resistant to type 1 interferons [64,65]. These findings indicate that signature sequence characteristics may provide a new opportunity to characterize which biological features of viruses increase their fitness for transmission.

### 2.4. Phylogenetic Inferences on Public Health Strategies to Control MSM and IDU Epidemics

Phylogenetic research has demonstrated that persons with recent infection drive transmission clustering among MSM and IDU groups; however, estimates are highly variable, representing 10 to 65% of all transmissions [26,27,63,66]. Discrepancies can be related to variations in HIV-1 prevalence (concentrated *vs.* diffuse epidemics), route of transmission (MSM vs. HET), sexual risk behaviors and depth of population sampling [29,41,49].

Studies in Quebec show a steady decline in IDU from 2002 to 2016, related in part to needle exchange programs, expanded access of ART, community-level declines in viral load. In contrast, rates of new infections among MSM have remained steady over this period. Phylogenetics reveal the decline in new infections associated with small cluster transmission networks have been offset by a relatively few persistent, self-staining and in some cases growing sub-epidemics (*n* = 30, cluster size 20–140) [27]. This has also been observed in the Netherlands MSM cohort [31].

Numerous groups have shown increased transmission efficiency and a disproportionate role of acute (0–6 weeks) and recent infection (first 6 months) in transmission dynamics [30,52,58]. Phylogenetics reveal how untimely diagnosis, particularly in younger populations can fuel transmission cascades. [52] These findings underscore how the inadequacies of current screening strategies can offset UN 90-90-90 treatment goals. The SPOT (2009–2016) and Actuel-sur-Rue (2012–2013) rapid testing sites in Montreal showed an improved ability to attract MSM (2% HIV^+^) [27,67]. The SPOT site revealed testing habits among MSM in the province with 50% of persons in Montreal reporting no prior test in the last year. Of note, testing frequency was inversely related to the number of reported partnerships in the three months prior to testing [27].

### 2.5. Genotypic Analysis Reveals Importance of Drug Regimen Selection in Long-Term HIV Management

**Figure 3:**
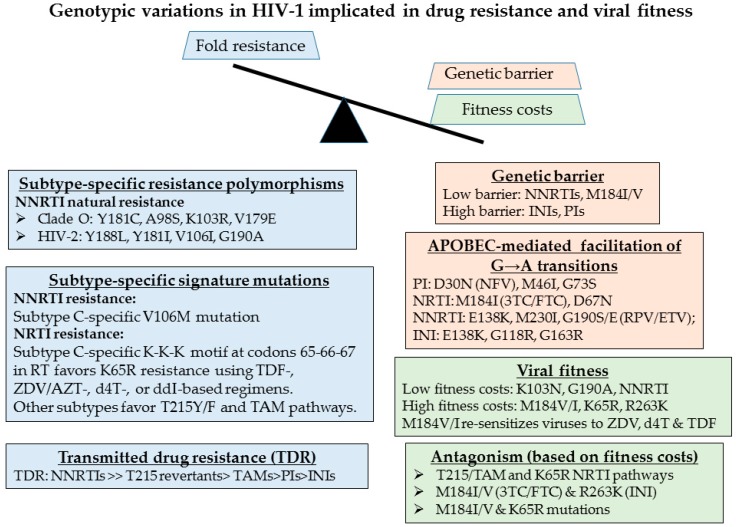
Signature genotypic features of HIV-1 variants that are implicated in the genetic barrier to development of resistance (orange), fold-resistance (blue) and viral replicative fitness (green). These include mutations implicated in the resistance to the nucleoside reverse transcriptase (RT) inhibitors (NRTIs), including tenofovir (TDF), zidovudine/AZT (ZDV), stavudine (d4T), didanosine (ddI), non-nucleoside RT inhibitors (NNRTIs), protease inhibitors (PIs), and integrase inhibitors (INIs).

The emergence of drug resistance can threaten the long-term benefit of ART regimens in resource-limited settings [14]. The evolution of drug resistance is multifactorial and is dependent on the genetic barrier, the fitness of the mutant variant, the level of resistance that a specific mutation confers and the frequency of that mutation within a given viral population (Figure 3) [17,68,69,70]. Drug resistance testing has enabled personalized strategies for the treatment of HIV-infected individuals in resource-rich settings. Baseline screening at diagnosis and prior to treatment initiation can identify transmitted drug resistance. Selection of treatment regimens can be guided by routine viral load (VL) testing and genotyping upon treatment failure (VL > 400 copies/mL).

An understanding of the impact of viral subtype in acquired and transmitted drug resistance are essential to guide the clinical management of HIV/AIDS in low- and middle-income settings where drug resistance testing is largely unaffordable [17,20,71]. The first-line therapies available in low- and middle-income setting often includes regimens that are no longer recommended in resource-rich settings. The NRTI components in the first-line ART options in low-income setting, often combine zidovudine (ZDV/AZT), stavudine (d4T) or didanosine (ddI) with lamivudine (3TC), as compared with high-income settings where tenofovir (TDF), or abacavir (ABC) are combined with 3TC or FTC [12,13,14]. The use of d4T-based regimens has been shown in many settings to be particularly prone to toxicity, treatment failure and the development of drug resistance. Switches of regimen in high-income settings are based on genotypic analysis to tailor regimens to individual needs as compared to resource limited settings where shifts are chosen by drug supply.

**Figure 4:**
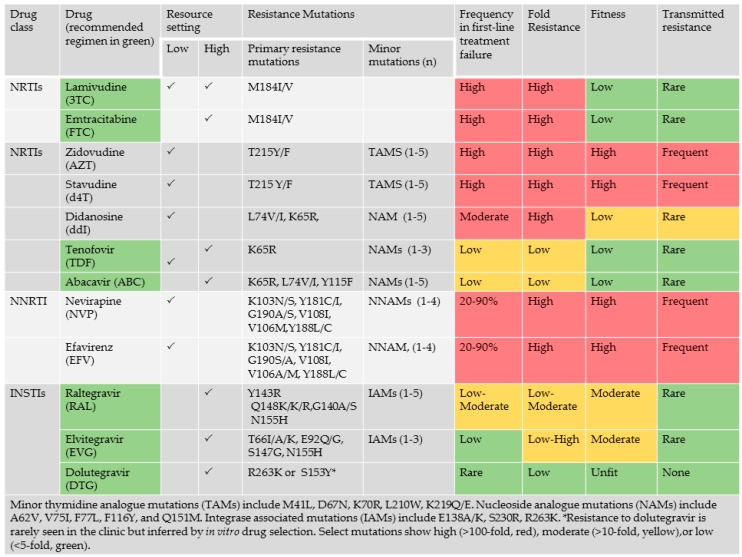
Impact of the choice of drugs in combination regimen on the development of drug resistance. The frequency of resistance in individuals failing first-line treatment, is influenced by the level (fold) resistance conferred by acquired mutations, the negative impact of resistance mutations on viral fitness.

HIV-1 genetic variation arises from repeated cycles of virus polymerization errors, recombination, APOBEC-mediated RNA editing and selective drug and immune pressure. The evolution of drug resistance is multifactorial, influenced by the genetic barrier to resistance, the replicative fitness and the level of resistance conferred by the acquired resistant variant (Figure 3). Viruses of the NNRTI drug class are replicatively fit with single point mutations conferring high level (>100-fold resistance). Select mutations, including Y181C/I, render group O and all strains of HIV-2 resistant to all drugs within the entire NNRTI class. Although the M184V/I point mutations confer high level resistance (>100 fold) to 3TC or FTC, an essential component most combination regimens, this mutation has high fitness costs, enhancing viral fidelity and resensitizing viruses to ZDV, d4T and TDF [72,73,74]. APOBEC-mediated G-to-A hypermutation—an ancient host defense mechanism responsible for lethal mutagenesis—facilitates the acquisition of select mutations against all drug classes [19,69] (Figure 4).

Recent studies reveal the high frequency of resistance mutations in West/Central and sub-Saharan Africa failing first-line therapy with NNRTI-based regimens after a 2 to 4-years period of treatment [12,13]. Most patients harbored resistance to nevirapine (NVP) (97.5%) or efavirenz (EFV) (75%), acquiring NNRTI -associated resistance mutations, including K103N, Y181C and G190A. There was a facilitated development of V106M in subtype C infections, related in part to a signature natural polymorphism at codon 106 [75]. The higher frequency of E138A, V106M, Y181C and H221Y in subtype C limit the utility of etravirine and rilpivirine in salvage regimens [12]. K65R was more common in subtype C, related to the signature K-K-K codon motif 64–66 codon motif [76,77]. The relatively low frequency of K65R may be related to the high fitness costs. The Q151M nucleoside analogue mutation observed 10% of subtype C isolates, negligible in other subtypes [12,13].

Recent survey data from the World Health Organization have documented an increase in the frequency of transmitted drug resistance (TDR) to NNRTIs related to the scale-up of NVP- or EFV-based regimens (http://www.who.int/hiv/pub/drugresistance/hivdr-report-2017). A rise of transmitted resistance to NNRTIs above 10% will offset the benefit of “treatment-as-prevention” strategies to end the pandemic by 2030. Indeed, 10% of genotyped treatment-naïve individuals acquired a virus resistant to nevirapine or efavirenz in 6 of 11 surveyed countries in sub Saharan Africa and South and Central America.

**Figure 5:**
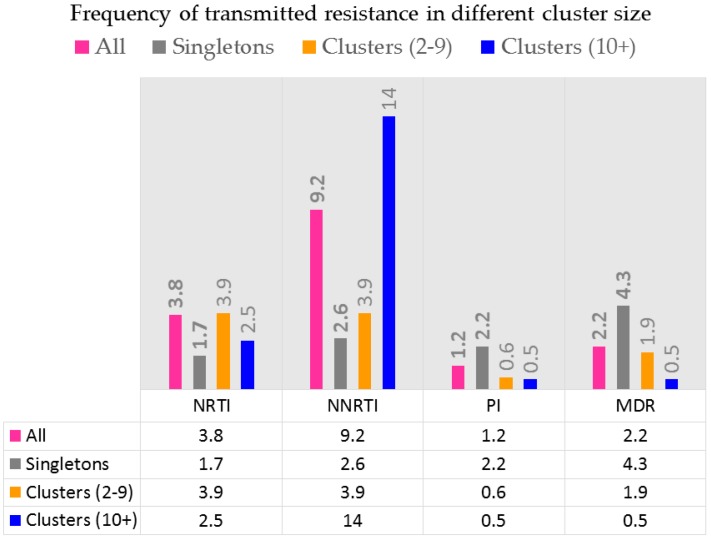
Transmitted resistance to various drug classes. Transmitted resistance to NNRTIs is related to viral fitness of viruses harboring K103N, G190A or Y181C point mutations. Transmitted resistance to NRTIs are related to viruses harboring M41L and revertants at codon 215.

The rise in TDR to NNRTIs may be more pronounced in MSM or IDU epidemics may also be related to transmission clustering [40,78]. As illustrated in Figure 5, transmission clustering has contributed to the selective spread of resistance to NNRTIs in Quebec. Indeed, the frequency of resistance to NNRTIs in treatment-naïve individuals has exceeded the frequency of resistance in NNRTI-experienced persons since 2009 following community-level declines in viral load associated with the rise in the use of TDF and emtricitabine (FTC) [40,79].

Transmitted resistance to NNRTIs arise due a relatively small number of NNRTI-resistance point mutations (K130N, Y181C, G190A and V106M) responsible for most cases of high-level resistance. This suggests that inexpensive point-mutation assays to detect these mutations may be useful for pre-therapy screening in regions with high levels of TDR [71]. A reliable point-of-care genotypic resistance test could identify which patients should receive standard first-line therapy and which should receive a protease-inhibitor-containing regimen.

### 2.6. The Need for Better Drugs in Africa

Cumulative findings suggest that the use of drugs with a low genetic barrier (e.g., NNRTIs) and high toxicity (e.g., d4T) have limited the efficacy and durability of the benefit of ART in sustaining long-term viral suppression.

Recent clinical trials have documented the superiority of triple drug regimens that include an INSTI over boosted protease inhibitors (PIs) or NNRTIs [80,81,82,83,84]. Current guidelines have shifted to INSTI-based regimens as the recommended treatment options for ART-naïve populations in resource-rich settings (http://aidsinfo.nih.gov/guidelines). Recent studies have shown the potential utility and cos-effectiveness of dolutegravir (DTG)- and elvitegravir (EVG)-based regimens in low- and middle-income countries [85,86,87,88].

DTG, the newest agent in the INSTI class, has advantages over other INSTIs [89]. Indeed, DTG was the first drug to out-perform EFV over 48 weeks in fully powered randomized trials [81,82]. DTG appears to be impervious to the development of drug resistance in “real-world” settings and this may have important ramifications in the future control of the HIV epidemic. The non-occurrence of drug resistance against DTG has extended to the nucleoside compounds within triple drug regimens [80,82,83,90]. This is noteworthy given that the M184V mutation, conferring resistance to 3TC and FTC is often the first to emerge in the aftermath of treatment failure.

This underscores the potential benefit of DTG as a cornerstone of HIV therapy in all countries in the world including those in sub-Saharan Africa and other developing countries [91,92]. The high barrier to resistance will likely maintain the durability of first-line regimens in settings in which genotypic resistance testing is unavailable. Successful prevention of HIV mother-to-child transmission with dolutegravir-based combination antiretroviral therapy has been observed in a vertically infected pregnant woman harboring multiclass highly drug-resistant HIV-1 [93]. The global shift from EFV-based to DTG-based regimens has begun in settings such as South Africa [91,92,94]. The high barrier to emergent resistance against DTG and INSTIs may mitigate the development of transmitted resistance compared to growing rates of resistance to EFV and other NNRTIs, ranging from 8 to 10% in different regional settings [40,71,95,96,97].

## 3. Conclusions

Stagnant HIV incidence rates emphasizes the importance of tailoring HIV interventions for select MSM, IDU and HET groups in different regional settings. Phylogenetics can be used to track disease and elucidate dynamic patterns of transmission. Most studies show that persons with undiagnosed recent infection drive onward transmission cascades. The failure to identify new infections in a timely fashion with early treatment intervention may partially explain the persistence of large cluster outbreaks. Public health interventions need to adequately address current screening strategies and linkage to prevention and care, particularly among younger populations. Indeed, there can be substantial overlap between persons in need for testing and those eligible for pre-exposure prophylaxis.

Phylogenetic strategies are advancing the concept of monitoring transmission networks in “real time” [28,34]. A high threshold will be needed to avoid falsely linking viral isolates within “active” transmission chains. Using phylogenetics to understand transmission patterns requires careful consideration of ethics, confidentiality and privacy. Similarly, understanding the impact of migration requires careful attention to avoid further discrimination in this group.

In the era of expanded ART coverage, it also remains imperative to provide the best treatment options available in low- and middle-income settings given the limited access to viral load and drug resistance screening. Newer integrase inhibitors, including dolutegravir and bictegravir appear to be impervious to the development of resistance in the clinic and the laboratory. Incorporation of integrase inhibitors into first-line regimens may be beneficial and cost-effective for the long-term management of HIV/AIDS and realizing “Getting-to-Zero” objectives.

## References

[1] Tebit D.M., Arts E.J. (2011). Tracking a century of global expansion and evolution of HIV to drive understanding and to combat disease. Lancet Infect. Dis..

[2] Spira S., Wainberg M.A., Loemba H., Turner D., Brenner B.G. (2003). Impact of clade diversity on HIV-1 virulence, antiretroviral drug sensitivity and drug resistance. J. Antimicrob. Chemother..

[3] Beyrer C., Sullivan P., Sanchez J., Baral S.D., Collins C., Wirtz A.L., Altman D., Trapence G., Mayer K. (2013). The increase in global HIV epidemics in MSM. AIDS.

[4] Wolf E., Herbeck J.T., van Rompaey S., Kitahata M., Thomas K., Pepper G., Frenkel L. (2017). Short Communication: Phylogenetic Evidence of HIV-1 Transmission Between Adult and Adolescent Men Who Have Sex with Men. AIDS Res. Hum. Retrovir..

[5] Magiorkinis G., Angelis K., Mamais I., Katzourakis A., Hatzakis A., Albert J., Lawyer G., Hamouda O., Struck D., Vercauteren J. (2016). The global spread of HIV-1 subtype B epidemic. Infect. Genet. Evol..

[6] Beloukas A., Psarris A., Giannelou P., Kostaki E., Hatzakis A., Paraskevis D. (2016). Molecular epidemiology of HIV-1 infection in Europe: An overview. Infect. Genet. Evol..

[7] Montaner J.S., Lima V.D., Barrios R., Yip B., Wood E., Kerr T., Shannon K., Harrigan P.R., Hogg R.S., Daly P. (2010). Association of highly active antiretroviral therapy coverage, population viral load and yearly new HIV diagnoses in British Columbia, Canada: A population-based study. Lancet.

[8] Jones A., Cremin I., Abdullah F., Idoko J., Cherutich P., Kilonzo N., Rees H., Hallett T., O’Reilly K., Koechlin F. (2014). Transformation of HIV from pandemic to low-endemic levels: A public health approach to combination prevention. Lancet.

[9] Quinn T.C., Wawer M.J., Sewankambo N., Serwadda D., Li C., Wabwire-Mangen F., Meehan M.O., Lutalo T., Gray R.H. (2000). Viral load and heterosexual transmission of human immunodeficiency virus type 1. Rakai Project Study Group. N. Engl. J. Med..

[10] Powers K.A., Kretzschmar M.E., Miller W.C., Cohen M.S. (2014). Impact of early-stage HIV transmission on treatment as prevention. Proc. Natl. Acad. Sci. USA.

[11] Cohen M.S., Chen Y.Q., McCauley M., Gamble T., Hosseinipour M.C., Kumarasamy N., Hakim J.G., Kumwenda J., Grinsztejn B., Pilotto J.H. (2011). Prevention of HIV-1 infection with early antiretroviral therapy. N. Engl. J. Med..

[12] Kityo C., Thompson J., Nankya I., Hoppe A., Ndashimye E., Warambwa C., Mambule I., van Oosterhout J.J., Wools-Kaloustian K., Bertagnolio S. (2017). Europe Africa Research Network for Evaluation of Second-line Therapy Trial, T.; HIV Drug Resistance Mutations in Non-B Subtypes After Prolonged Virological Failure on NNRTI-Based First-Line Regimens in Sub-Saharan Africa. J. Acquir. Immune Defic. Syndr..

[13] Villabona-Arenas C.J., Vidal N., Guichet E., Serrano L., Delaporte E., Gascuel O., Peeters M. (2016). In-depth analysis of HIV-1 drug resistance mutations in HIV-infected individuals failing first-line regimens in West and Central Africa. AIDS.

[14] Brenner B., Wainberg M.A. (2016). We need to use the best antiretroviral drugs worldwide to prevent HIV drug resistance. AIDS.

[15] Brenner B.G., Wainberg M.A. (2017). Clinical benefit of dolutegravir in HIV-1 management related to the high genetic barrier to drug resistance. Virus Res..

[16] Beyrer C., Pozniak A. (2017). HIV Drug Resistance—An Emerging Threat to Epidemic Control. N. Engl. J. Med..

[17] Wainberg M.A., Zaharatos G.J., Brenner B.G. (2011). Development of antiretroviral drug resistance. N. Engl. J. Med..

[18] Kantor R., Katzenstein D.A., Efron B., Carvalho A.P., Wynhoven B., Cane P., Clarke J., Sirivichayakul S., Soares M.A., Snoeck J. (2005). Impact of HIV-1 subtype and antiretroviral therapy on protease and reverse transcriptase genotype: Results of a global collaboration. PLoS Med..

[19] Rhee S.Y., Sankaran K., Varghese V., Winters M.A., Hurt C.B., Eron J.J., Parkin N., Holmes S.P., Holodniy M., Shafer R.W. (2016). HIV-1 Protease, Reverse Transcriptase and Integrase Variation. J. Virol..

[20] Rhee S.Y., Varghese V., Holmes S.P., van Zyl G.U., Steegen K., Boyd M.A., Cooper D.A., Nsanzimana S., Saravanan S., Charpentier C. (2017). Mutational Correlates of Virological Failure in Individuals Receiving a WHO-Recommended Tenofovir-Containing First-Line Regimen: An International Collaboration. EBioMedicine.

[21] Rhee S.Y., Fessel W.J., Liu T.F., Marlowe N.M., Rowland C.M., Rode R.A., Vandamme A.M., van Laethem K., Brun-Vezinet F., Calvez V. (2009). Predictive value of HIV-1 genotypic resistance test interpretation algorithms. J. Infect. Dis..

[22] Granich R., Crowley S., Vitoria M., Smyth C., Kahn J.G., Bennett R., Lo Y.R., Souteyrand Y., Williams B. (2010). Highly active antiretroviral treatment as prevention of HIV transmission: Review of scientific evidence and update. Curr. Opin. HIV AIDS.

[23] DeHovitz J., Uuskula A., El-Bassel N. (2014). The HIV epidemic in Eastern Europe and Central Asia. Curr. HIV/AIDS Rep..

[24] El-Bassel N., Shaw S.A., Dasgupta A., Strathdee S.A. (2014). Drug use as a driver of HIV risks: Re-emerging and emerging issues. Curr. Opin. HIV AIDS.

[25] Lebouche B., Engler K., Levy J.J., Gilmore N., Spire B., Rozenbaum W., Lacene T., Routy J.P. (2014). French HIV experts on early antiretroviral treatment for prevention: Uncertainty and heterogeneity. J. Int. Assoc. Provid. AIDS Care.

[26] Liu A.Y., Buchbinder S.P. (2017). CROI 2017: HIV Epidemic Trends and Advances in Prevention. Top. Antivir. Med..

[27] Brenner B.G., Ibanescu R.I., Hardy I., Stephens D., Otis J., Moodie E., Grossman Z., Vandamme A.M., Roger M., Wainberg M.A. (2017). Large cluster outbreaks sustain the HIV epidemic among MSM in Quebec. AIDS.

[28] Poon A.F., Gustafson R., Daly P., Zerr L., Demlow S.E., Wong J., Woods C.K., Hogg R.S., Krajden M., Moore D. (2016). Near real-time monitoring of HIV transmission hotspots from routine HIV genotyping: An implementation case study. Lancet HIV.

[29] Brenner B., Wainberg M.A., Roger M. (2013). Phylogenetic inferences on HIV-1 transmission: Implications for the design of prevention and treatment interventions. AIDS.

[30] Brenner B.G., Roger M., Routy J.P., Moisi D., Ntemgwa M., Matte C., Baril J.G., Thomas R., Rouleau D., Bruneau J. (2007). High rates of forward transmission events after acute/early HIV-1 infection. J. Infect. Dis..

[31] Bezemer D., Cori A., Ratmann O., van Sighem A., Hermanides H.S., Dutilh B.E., Gras L., Rodrigues Faria N., van den Hengel R., Duits A.J. (2015). Dispersion of the HIV-1 Epidemic in Men Who Have Sex with Men in the Netherlands: A Combined Mathematical Model and Phylogenetic Analysis. PLoS Med..

[32] Bezemer D., Faria N.R., Hassan A., Hamers R.L., Mutua G., Anzala O., Mandaliya K., Cane P., Berkley J.A., Rinke de Wit T.F. (2014). HIV Type 1 transmission networks among men having sex with men and heterosexuals in Kenya. AIDS Res. Hum. Retrovir..

[33] Ratmann O., Hodcroft E.B., Pickles M., Cori A., Hall M., Lycett S., Colijn C., Dearlove B., Didelot X., Frost S. (2017). Phylogenetic Tools for Generalized HIV-1 Epidemics: Findings from the PANGEA-HIV Methods Comparison. Mol. Biol. Evol..

[34] Ratmann O., van Sighem A., Bezemer D., Gavryushkina A., Jurriaans S., Wensing A., de Wolf F., Reiss P., Fraser C. (2016). Sources of HIV infection among men having sex with men and implications for prevention. Sci. Transl. Med..

[35] Kouyos R.D., von Wyl V., Yerly S., Boni J., Taffe P., Shah C., Burgisser P., Klimkait T., Weber R., Hirschel B. (2010). Molecular epidemiology reveals long-term changes in HIV type 1 subtype B transmission in Switzerland. J. Infect. Dis..

[36] Marzel A., Shilaih M., Yang W.L., Boni J., Yerly S., Klimkait T., Aubert V., Braun D.L., Calmy A., Furrer H. (2016). HIV-1 Transmission During Recent Infection and During Treatment Interruptions as Major Drivers of New Infections in the Swiss HIV Cohort Study. Clin. Infect. Dis..

[37] Shilaih M., Marzel A., Yang W.L., Scherrer A.U., Schupbach J., Boni J., Yerly S., Hirsch H.H., Aubert V., Cavassini M. (2016). Genotypic Resistance Tests Sequences Reveal the Role of Marginalized Populations in HIV-1 Transmission in Switzerland. Sci. Rep..

[38] Villandre L., Stephens D.A., Labbe A., Gunthard H.F., Kouyos R., Stadler T., Swiss HIV Cohort Study (2016). Assessment of Overlap of Phylogenetic Transmission Clusters and Communities in Simple Sexual Contact Networks: Applications to HIV-1. PLoS ONE.

[39] Brenner B.G., Roger M., Stephens D., Moisi D., Hardy I., Weinberg J., Turgel R., Charest H., Koopman J., Wainberg M.A. (2011). Transmission clustering drives the onward spread of the HIV epidemic among men who have sex with men in Quebec. J. Infect. Dis..

[40] Brenner B.G., Roger M., Moisi D.D., Oliveira M., Hardy I., Turgel R., Charest H., Routy J.P., Wainberg M.A. (2008). Transmission networks of drug resistance acquired in primary/early stage HIV infection. AIDS.

[41] Brenner B.G., Wainberg M.A. (2013). Future of phylogeny in HIV prevention. J. Acquir. Immune Defic. Syndr..

[42] Bezemer D., van Sighem A., Lukashov V.V., van der Hoek L., Back N., Schuurman R., Boucher C.A., Claas E.C., Boerlijst M.C., Coutinho R.A. (2010). Transmission networks of HIV-1 among men having sex with men in the Netherlands. AIDS.

[43] Leigh Brown A.J., Lycett S.J., Weinert L., Hughes G.J., Fearnhill E., Dunn D.T. (2011). Transmission network parameters estimated from HIV sequences for a nationwide epidemic. J. Infect. Dis..

[44] Wainberg M.A., Mesplede T., Raffi F. (2013). What if HIV were unable to develop resistance against a new therapeutic agent?. BMC Med..

[45] Wainberg M.A., Friedland G. (1998). Public health implications of antiretroviral therapy and HIV drug resistance. JAMA.

[46] German D., Grabowski M.K., Beyrer C. (2016). Enhanced use of phylogenetic data to inform public health approaches to HIV among men who have sex with men. Sex. Health.

[47] Brenner BG I.R., Roger M., Oliveira M., Hardy I., Wainberg M.A. Phylogenetic, Epidemiological and Virological Insights on the Rise of Large Cluster Outbreaks Fueling the HIV-1 Epidemic among Men Having Sex with Men within Quebec. Proceedings of the 12th International Workshop on HIV Transmission-Principles of Intervention;.

[48] Esbjornsson J., Mild M., Audelin A., Fonager J., Skar H., Bruun Jorgensen L., Liitsola K., Bjorkman P., Bratt G., Gisslen M. (2016). HIV-1 transmission between MSM and heterosexuals and increasing proportions of circulating recombinant forms in the Nordic Countries. Virus Evol..

[49] Hassan A.S., Pybus O.G., Sanders E.J., Albert J., Esbjornsson J. (2017). Defining HIV-1 transmission clusters based on sequence data. AIDS.

[50] Paraskevis D., Nikolopoulos G., Tsiara C., Paraskeva D., Antoniadou A., Lazanas M., Gargalianos P., Psychogiou M., Malliori M., Kremastinou J. (2011). HIV-1 outbreak among injecting drug users in Greece, 2011: A preliminary report. Euro Surveill..

[51] Yebra G., Ragonnet-Cronin M., Ssemwanga D., Parry C.M., Logue C.H., Cane P.A., Kaleebu P., Brown A.J. (2015). Analysis of the history and spread of HIV-1 in Uganda using phylodynamics. J. Gen. Virol..

[52] Hue S., Brown A.E., Ragonnet-Cronin M., Lycett S.J., Dunn D.T., Fearnhill E., Dolling D.I., Pozniak A., Pillay D., Delpech V.C. (2014). Phylogenetic analyses reveal HIV-1 infections between men misclassified as heterosexual transmissions. AIDS.

[53] Ragonnet-Cronin M., Lycett S.J., Hodcroft E.B., Hue S., Fearnhill E., Brown A.E., Delpech V., Dunn D., Leigh Brown A.J., United Kingdom HIV Drug Resistance Database (2016). Transmission of Non-B HIV Subtypes in the United Kingdom Is Increasingly Driven by Large Non-Heterosexual Transmission Clusters. J. Infect. Dis..

[54] Patel P., Borkowf C.B., Brooks J.T., Lasry A., Lansky A., Mermin J. (2014). Estimating per-act HIV transmission risk: A systematic review. AIDS.

[55] Baeten J.M., Overbaugh J. (2003). Measuring the infectiousness of persons with HIV-1: Opportunities for preventing sexual HIV-1 transmission. Curr. HIV Res..

[56] Keele B.F., Giorgi E.E., Salazar-Gonzalez J.F., Decker J.M., Pham K.T., Salazar M.G., Sun C., Grayson T., Wang S., Li H. (2008). Identification and characterization of transmitted and early founder virus envelopes in primary HIV-1 infection. Proc. Natl. Acad. Sci. USA.

[57] Salazar-Gonzalez J.F., Bailes E., Pham K.T., Salazar M.G., Guffey M.B., Keele B.F., Derdeyn C.A., Farmer P., Hunter E., Allen S. (2008). Deciphering human immunodeficiency virus type 1 transmission and early envelope diversification by single-genome amplification and sequencing. J. Virol..

[58] Cohen M.S., Shaw G.M., McMichael A.J., Haynes B.F. (2011). Acute HIV-1 Infection. N. Engl. J. Med..

[59] Gottlieb G.S., Heath L., Nickle D.C., Wong K.G., Leach S.E., Jacobs B., Gezahegne S., van’t Wout A.B., Jacobson L.P., Margolick J.B. (2008). HIV-1 variation before seroconversion in men who have sex with men: Analysis of acute/early HIV infection in the multicenter AIDS cohort study. J. Infect. Dis..

[60] Vrbik I., Stephens D.A., Roger M., Brenner B.G. (2015). The Gap Procedure: For the identification of phylogenetic clusters in HIV-1 sequence data. BMC Bioinform..

[61] Lessells R.J., Stott K.E., Manasa J., Naidu K.K., Skingsley A., Rossouw T., de Oliveira T. (2014). Implementing antiretroviral resistance testing in a primary health care HIV treatment programme in rural KwaZulu-Natal, South Africa: Early experiences, achievements and challenges. BMC Health Serv. Res..

[62] Lessells R.J., Avalos A., de Oliveira T. (2013). Implementing HIV-1 genotypic resistance testing in antiretroviral therapy programs in Africa: Needs, opportunities and challenges. AIDS Rev..

[63] Brenner B.G., Ibanescu R.I., Oliveira M., Roger M., Hardy I., Routy J.P., Kyeyune F., Quinones-Mateu M.E., Wainberg M.A., Montreal PHI Cohort Study Group (2017). HIV-1 strains belonging to large phylogenetic clusters show accelerated escape from integrase inhibitors in cell culture compared with viral isolates from singleton/small clusters. J. Antimicrob. Chemother..

[64] Joseph S.B., Swanstrom R., Kashuba A.D., Cohen M.S. (2015). Bottlenecks in HIV-1 transmission: Insights from the study of founder viruses. Nat. Rev. Microbiol..

[65] Iyer S.S., Bibollet-Ruche F., Sherrill-Mix S., Learn G.H., Plenderleith L., Smith A.G., Barbian H.J., Russell R.M., Gondim M.V., Bahari C.Y. (2017). Resistance to type 1 interferons is a major determinant of HIV-1 transmission fitness. Proc. Natl. Acad. Sci. USA.

[66] Buchbinder S.P., Liu A.Y. (2016). CROI 2016: Hot Spots in HIV Infection and Advances in HIV Prevention. Top. Antivir. Med..

[67] Engler K., Rollet K., Lessard D., Thomas R., Lebouche B. (2016). Ability of a rapid HIV testing site to attract and test vulnerable populations: A cross-sectional study on Actuel sur Rue. Int. J. STD AIDS.

[68] Brenner B.G., Thomas R., Blanco J.L., Ibanescu R.I., Oliveira M., Mesplede T., Golubkov O., Roger M., Garcia F., Martinez E. (2016). Development of a G118R mutation in HIV-1 integrase following a switch to dolutegravir monotherapy leading to cross-resistance to integrase inhibitors. J. Antimicrob. Chemother..

[69] Palanisamy N., Osman N., Ohnona F., Xu H.T., Brenner B., Mesplede T., Wainberg M.A. (2017). Does antiretroviral treatment change HIV-1 codon usage patterns in its genes: A preliminary bioinformatics study. AIDS Res. Ther..

[70] Brenner B.G., Lowe M., Moisi D., Hardy I., Gagnon S., Charest H., Baril J.G., Wainberg M.A., Roger M. (2011). Subtype diversity associated with the development of HIV-1 resistance to integrase inhibitors. J. Med. Virol..

[71] Rhee S.Y., Blanco J.L., Jordan M.R., Taylor J., Lemey P., Varghese V., Hamers R.L., Bertagnolio S., Rinke de Wit T.F., Aghokeng A.F. (2015). Geographic and temporal trends in the molecular epidemiology and genetic mechanisms of transmitted HIV-1 drug resistance: An individual-patient- and sequence-level meta-analysis. PLoS Med..

[72] Wainberg M.A., Drosopoulos W.C., Salomon H., Hsu M., Borkow G., Parniak M., Gu Z., Song Q., Manne J., Islam S. (1996). Enhanced fidelity of 3TC-selected mutant HIV-1 reverse transcriptase. Science.

[73] Petrella M., Wainberg M.A. (2002). Might the M184V substitution in HIV-1 RT confer clinical benefit?. AIDS Rev..

[74] Turner D., Brenner B.G., Routy J.P., Petrella M., Wainberg M.A. (2004). Rationale for maintenance of the M184v resistance mutation in human immunodeficiency virus type 1 reverse transcriptase in treatment experienced patients. New Microbiol..

[75] Brenner B., Turner D., Oliveira M., Moisi D., Detorio M., Carobene M., Marlink R.G., Schapiro J., Roger M., Wainberg M.A. (2003). A V106M mutation in HIV-1 clade C viruses exposed to efavirenz confers cross-resistance to non-nucleoside reverse transcriptase inhibitors. AIDS.

[76] Brenner B.G., Coutsinos D. (2009). The K65R mutation in HIV-1 reverse transcriptase: Genetic barriers, resistance profile and clinical implications. HIV Ther..

[77] Brenner B.G., Oliveira M., Doualla-Bell F., Moisi D.D., Ntemgwa M., Frankel F., Essex M., Wainberg M.A. (2006). HIV-1 subtype C viruses rapidly develop K65R resistance to tenofovir in cell culture. AIDS.

[78] Yerly S., Junier T., Gayet-Ageron A., Amari E.B., von Wyl V., Gunthard H.F., Hirschel B., Zdobnov E., Kaiser L. (2009). The impact of transmission clusters on primary drug resistance in newly diagnosed HIV-1 infection. AIDS.

[79] Charest H., Doualla-Bell F., Cantin R., Murphy D.G., Lemieux L., Brenner B., Hardy I., Moisi D., Lo E., Baril J.G. (2014). A Significant Reduction in the Frequency of HIV-1 Drug Resistance in Quebec from 2001 to 2011 Is Associated with a Decrease in the Monitored Viral Load. PLoS ONE.

[80] Raffi F., Jaeger H., Quiros-Roldan E., Albrecht H., Belonosova E., Gatell J.M., Baril J.G., Domingo P., Brennan C., Almond S. (2013). Once-daily dolutegravir versus twice-daily raltegravir in antiretroviral-naive adults with HIV-1 infection (SPRING-2 study): 96 Week results from a randomised, double-blind, non-inferiority trial. Lancet Infect. Dis..

[81] Walmsley S.L., Antela A., Clumeck N., Duiculescu D., Eberhard A., Gutierrez F., Hocqueloux L., Maggiolo F., Sandkovsky U., Granier C. (2013). Dolutegravir plus abacavir-lamivudine for the treatment of HIV-1 infection. N. Engl. J. Med..

[82] Walmsley S., Baumgarten A., Berenguer J., Felizarta F., Florence E., Khuong-Josses M.A., Kilby J.M., Lutz T., Podzamczer D., Portilla J. (2015). Brief Report: Dolutegravir Plus Abacavir/Lamivudine for the Treatment of HIV-1 Infection in Antiretroviral Therapy-Naive Patients: Week 96 and Week 144 Results from the SINGLE Randomized Clinical Trial. J. Acquir. Immune Defic. Syndr..

[83] Clotet B., Feinberg J., van Lunzen J., Khuong-Josses M.A., Antinori A., Dumitru I., Pokrovskiy V., Fehr J., Ortiz R., Saag M. (2014). Once-daily dolutegravir versus darunavir plus ritonavir in antiretroviral-naive adults with HIV-1 infection (FLAMINGO): 48 Week results from the randomised open-label phase 3b study. Lancet.

[84] Miller M.M., Liedtke M.D., Lockhart S.M., Rathbun R.C. (2015). The role of dolutegravir in the management of HIV infection. Infect. Drug Resist..

[85] Squires K., Kityo C., Hodder S., Johnson M., Voronin E., Hagins D., Avihingsanon A., Koenig E., Jiang S., White K. (2016). Integrase inhibitor versus protease inhibitor based regimen for HIV-1 infected women (WAVES): A randomised, controlled, double-blind, phase 3 study. Lancet HIV.

[86] Kaplan R., Wood R. (2017). Resistance to first-line ART and a role for dolutegravir. Lancet HIV.

[87] Phillips A.N., Cambiano V., Nakagawa F., Revill P., Jordan M.R., Hallett T.B., Doherty M., De Luca A., Lundgren J.D., Mhangara M. (2017). Cost-effectiveness of public-health policy options in the presence of pretreatment NNRTI drug resistance in sub-Saharan Africa: A modelling study. Lancet HIV.

[88] Venter W.D.F., Clayden P., Serenata C., Consortium O. (2017). The ADVANCE study: A groundbreaking trial to evaluate a candidate universal antiretroviral regimen. Curr. Opin. HIV AIDS.

[89] Anstett K., Brenner B., Mesplede T., Wainberg M.A. (2017). HIV drug resistance against strand transfer integrase inhibitors. Retrovirology.

[90] Raffi F., Rachlis A., Brinson C., Arasteh K., Gorgolas M., Brennan C., Pappa K., Almond S., Granier C., Nichols W.G. (2015). Dolutegravir efficacy at 48 weeks in key subgroups of treatment-naive HIV-infected individuals in three randomized trials. AIDS.

[91] Cohn J., Bekker L.G., Bygrave H., Calmy A. (2015). Hit me with your best shot: Dolutegravir—A space in the next WHO guidelines?. AIDS.

[92] Wainberg M.A., Mesplede T. (2015). Implications for the future of the HIV epidemic if drug resistance against dolutegravir cannot occur in first-line therapy. J. Int. AIDS Soc..

[93] Pinnetti C., Tintoni M., Ammassari A., Tamburrini E., Bernardi S., Liuzzi G., Scambia G., Perno C.F., Floridia M., Antinori A. (2015). Successful prevention of HIV mother-to-child transmission with dolutegravir-based combination antiretroviral therapy in a vertically infected pregnant woman with multiclass highly drug-resistant HIV-1. AIDS.

[94] Raffi F., Pozniak A.L., Wainberg M.A. (2014). Has the time come to abandon efavirenz for first-line antiretroviral therapy?. J. Antimicrob. Chemother..

[95] Hofstra L.M., Sauvageot N., Albert J., Alexiev I., Garcia F., Struck D., van de Vijver D.A., Asjo B., Beshkov D., Coughlan S. (2016). Transmission of HIV Drug Resistance and the Predicted Effect on Current First-line Regimens in Europe. Clin. Infect. Dis..

[96] Hamers R.L., Wallis C.L., Kityo C., Siwale M., Mandaliya K., Conradie F., Botes M.E., Wellington M., Osibogun A., Sigaloff K.C. (2011). HIV-1 drug resistance in antiretroviral-naive individuals in sub-Saharan Africa after rollout of antiretroviral therapy: A multicentre observational study. Lancet Infect. Dis..

[97] Ceccherini-Silberstein F., van Baelen K., Armenia D., Trignetti M., Rondelez E., Fabeni L., Scopelliti F., Pollicita M., van Wesenbeeck L., van Eygen V. (2010). Secondary integrase resistance mutations found in HIV-1 minority quasispecies in integrase therapy-naive patients have little or no effect on susceptibility to integrase inhibitors. Antimicrob. Agents Chemother..

